# Influence of divergence in residual feed intake on growth performance, carcass traits, meat quality, muscle fiber morphology, and blood chemistry in indigenous chickens

**DOI:** 10.1016/j.psj.2025.105712

**Published:** 2025-08-20

**Authors:** Wenjing Chen, Weiqi Wang, Xin Wang, Xuling Liu, Liang Chang, Haoming Chang, Yunxia He, Zhaoyu Geng, Sihua Jin

**Affiliations:** a College of Animal Science and Technology, Anhui Agricultural University, Hefei 230036, China; b Anhui Provincial Key Laboratory of Local Animal Genetic Resources Conservation and Bio-breeding, Hefei 230036, China

**Keywords:** Residual feed intake, Chicken, Growth performance, Blood biochemical parameters, Correlation analysis

## Abstract

Feed efficiency (**FE**) is an important factor restricting development of the poultry industries. Residual feed intake (**RFI**) serves as an indicator for measuring FE in breeding initiatives. This study aimed to explore the influence of divergence in RFI on growth performance, carcass traits, meat quality, muscle fiber morphology, and blood chemistry in indigenous chickens. A total of 500 one-day-old male chickens of similar body weight had RFI assessed at 70–120 d. Twenty high RFI (**HRFI**) and 20 low RFI (**LRFI**) chickens were evaluated for carcass traits, meat quality, muscle fiber morphology, and blood biochemical parameters. The results showed that although RFI was significantly positively correlated with average daily gain, average daily feed intake (**ADFI**), and feed conversion ratio (**FCR**) (*P*<0.01), no significant correlation existed between the initial, final, and metabolic body weight. Concerning slaughter performance, abdominal fat yield (**AFY**) and weight (**AFW**) were lower in the LRFI group (*P*<0.01). Regarding meat quality, the pH_24h_ of the thigh muscle and the shear force of the breast muscle were lower in LRFI group (*P*<0.01). Muscle fibers did not differ between the HRFI and LRFI groups. Regarding blood biochemical parameters, non-esterified fatty acid, creatinine (**CREA**), low-density lipoprotein cholesterol (**LDL-C**), and cholesterol (**CHOL**) levels were higher in the HRFI group (*P* < 0.05). Correlation analysis suggested that RFI was positively correlated with FCR and ADFI (*P* < 0.01). RFI had a significant positive effect on AFY and AFW (*P* < 0.01). Moreover, RFI demonstrated a positive effect on the pH_24h_ of the thigh muscle and the shear force of the breast muscle (*P* < 0.01). Furthermore, LDL-C, CHOL, and CREA levels were positively correlated with RFI (*P* < 0.05). In summary, low RFI within a tolerable range decreases fat deposition and increases FE without impacting production performance. LDL-C, CHOL, and CREA are indirect indicators of FE. This study offers significant insights into the biological processes underlying variability in RFI in indigenous chickens.

## Introduction

Feed consumption represents the largest cost in livestock husbandry, significantly impacting production (Romanzin et al., 2024). In poultry, feed expenses account for approximately 70 % of total production costs (Willems et al., 2013). As land resources continue to decrease, competition between humans and livestock for arable land intensifies, making improvements in feed efficiency (**FE**) essential for reducing costs and minimizing environmental impact (Anand et al., 2021). Current poultry breeding efforts focus on enhancing FE without compromising performance (Lin and Aggrey, 2013). The feed conversion ratio (**FCR**) and residual feed intake (**RFI**) are the two primary metrics used to measure FE (Aboshady et al., 2024). Of these metrics, FCR is a characteristic that is moderately heritable (O'Sullivan et al., 1992). As a proportional measure, FCR, which represents the ratio of feed consumption to weight gain, lacks direct statistical significance (Xu et al., 2014; Yi et al., 2018). In contrast, RFI, first introduced by Koch in 1963, measures the difference between predicted and actual feed intake (Koch et al., 1963) and is now the preferred index for measuring FE in poultry (Metzler-Zebeli et al., 2019).

Maintaining superior growth performance, meat quality, and carcass traits in chicken is crucial from the perspective of the consumer. Studying muscle fiber histology helps determine the meat quality and palatability of livestock (Yu et al., 2018). These key economic traits not only impact production efficiency but also align with consumer demands, promoting sustainable poultry farming. Therefore, they should be considered before incorporating the RFI in breeding programs. While research suggests that RFI can be integrated into commercial breeding selection indices (Willems et al., 2019), it usually takes considerable time and labor to select chickens with high FE. Previous studies on the FE have confirmed that blood plasma metabolites are related to energy requirements (Cônsolo et al., 2018). Moreover, blood parameters may help identify variations in feed utilization efficiency by revealing physiological metabolic responses (Leão et al., 2021).

China possesses a diverse range of indigenous chicken breeds, yet research on them remains limited. The Huaibei Partridge chicken, native to Suzhou, Anhui province, China, is popular for its tolerance to roughage, tender meat, delicious taste, early maturation, and adaptability to high-temperature and humid climates. However, consistent with commercial broilers, indigenous chicken breeds struggle with low FE. Therefore, the purpose of this study was to evaluate how RFI divergence affected growth performance, carcass traits, meat quality, muscle fiber morphology, and blood biochemical markers in Huaibei Partridge chicken. The important insights provided by our current research can help direct the selection of economically advantageous features for indigenous chicken breeds.

## Materials and methods

### Ethics statement

The Animal Care and Use Committee approved this research in compliance with ethical guidelines (approval number: SYXK 2016−007). The study adhered to regulations outlined in the Administration of Affairs Concerning Experimental Animals, which was revised by China's Ministry of Science and Technology in June 2004.

### Animals, diets, and experimental procedures

Huaibei Partridge chickens were supplied and raised by Xiangfeng Poultry Conservation and Breeding Co, Ltd. (Anhui, China), which provided complete pedigree information. All chickens were individually weighed, sexed, and wing banded on the day of hatching, before being raised indoors. From day 1, they were housed together and fed collectively until day 70. A total of 500 male chickens with similar body weight (703.2 ± 59.6 g) were chosen at 70 d of age and moved to separate cages. Following the company′s management protocols, all birds were raised under uniform environmental and management conditions. All experimental chickens were provided with water and feed *ad libitum*. Their compositions are presented in Table 1.

**Table 1:** Ingredients and nutrient levels of experimental diets (as-fed basis).

Items	1-30 d	31-120 d
Ingredients (%)		
Corn	67.35	69.25
Soybean meal	21.00	19.00
Wheat bran	6.07	6.38
Soybean oil	2.50	2.60
Choline chloride	0.12	0.12
NaCl	0.16	0.15
Calcium hydrophosphate	0.50	0.30
Limestone	1.30	1.20
Premix^1^	1.00	1.00
Total	100.00	100.00
Nutrient levels (%)		
Metabolic energy (MJ*kg^−1^)	12.40	12.68
Crude protein	19.00	17.50
Calcium	0.85	0.70
Available phosphorus	0.35	0.35
Lysine	0.95	0.90
Methionine	0.38	0.36
Threonine	0.64	0.56

^1^ Premix provided per kg of diet: vitamin A, 8 500 IU; vitamin D_3_, 3 000 IU; vitamin E, 20 IU; vitamin K_3_, 2.5 mg; vitamin B_1_, 2.8 mg; vitamin B_2_, 7.0 mg; vitamin B_6_, 1.50 mg; vitamin B_12_, 0.03 mg; biotin, 0.25 mg; nicotinic acid, 40.0 mg; pantothenic acid, 30.0 mg; NaHCO_3_, 1 050 mg; Cu, 8.0 mg; Fe, 85.0 mg; Mn, 61.0 mg; Se, 0.15 mg; I, 0.35 mg; Zn, 50.0 mg.

### RFI determination and ranking

Body weight (**BW**) measurements were made at 70 and 120 d of age. Feed intake and average daily feed intake (**ADFI**) were recorded, and the ADG, FCR, and metabolic BW (**MBW^0.75^**) were calculated for the 70 – 120 d period. The following formula was used to determine the RFI:

$$\text{RFI}=\text{ADFI}-(c_{0}+c_{1}*\text{MB}W^{0.75}+c_{2}*\text{ADG})$$

where c_0_ denotes the intercept, and c_1_ and c_2_ are the respective partial regression coefficients. Chickens were ranked by RFI values and divided into two groups: high RFI (HRFI ≥ mean + 0.5*SD) and low RFI (LRFI ≤ mean−0.5*SD). Twenty chickens from both the HRFI and LRFI groups were chosen for further research.

### Carcass traits and meat quality measurements

After a 12-h fast, 40 chickens from each of the two RFI groups were weighed to determine their live weight (**LW**) at the conclusion of the feeding experiment and then killed. Carcass weight (**CW**) included the plucked body with the head and feet. Semi-eviscerated weight (**SEW**) was calculated by subtracting the intestines, windpipe, pancreas, esophagus, crop, spleen, reproductive organs, stomach contents, and cuticular membranes from CW. Eviscerated weight (**EW**) was obtained by further removing the heart, liver, gizzard, glandular stomach, muscular stomach, lungs, and abdominal fat. Abdominal fat, thigh muscle, and breast muscle were then separated and weighed. The formula for calculating the yield of carcass weight was (CW/LW) × 100 %. The SEW yield was calculated as (SEW/LW)×100 %, and the EW yield was calculated as (EW/LW)×100 %. The thigh weight yield was determined as (bilateral of thigh muscle weight/EW) ×100 %, while the breast muscle weight yield was determined as (bilateral of breast muscle weight/EW)×100 %.

The right-side breast and thigh muscles were selected for meat quality assessment. This involved determining the pH value after 45 min (pH_45min_), pH value after 24 h (pH_24h_), meat color, shear force for each chicken, and calculating the cooking loss. A pH meter (PHSJ-5, Shanghai INESA Scientific Instrument Co., Ltd., Shanghai, China) was utilized to measure pH_45min_ and pH_24h_ with the muscle stored at 4°C. Prior to and during pH measurements, the pH meter was calibrated using phosphate buffers with pH values of 4.01 and 7.01. The pH values were measured at three locations after 45 min and 24 h. The color of the meat was evaluated using a Chroma Meter CR-300 (Konica Minolta, Tokyo, Japan); lightness (L*), redness (a*), and yellowness (b*) were measured from three different directions positioned parallel to and close to the muscle surface. The final color value was calculated as the average of these measurements. The method for the cooking loss of breast and thigh muscles was in accordance with the standard issued by the Ministry of Agriculture and Rural Affairs of the People’s Republic of China (2015). The muscle samples were weighed (**W0**), sealed in plastic bags, and heated at 75°C until the center temperature reached 70°C, then the heating was stopped. Once cooled to room temperature, the samples were gently blotted dry with a paper towel instead of being squeezed and then reweighed (**W**). The cooking loss was computed using the following equation:

Cookingloss(%)=(W−W0)/W0×100%

For shear force analysis, cooked breast and thigh meat were sliced along muscle fibers into strips measuring 3 cm in length, 1 cm in width, and 1 cm in thickness, following the standard proposed by the Ministry of Agriculture and Rural Affairs of the People’s Republic of China (2015). Shear force was measured using a TenovoC-LM3B device (Tenovo International Co., Ltd., Beijing, China).

### Muscle fiber morphology

From each group, 20 chickens were chosen at random for slaughter (HRFI: *n* = 20; LRFI: *n* = 20). Breast and thigh muscles were collected from the central portion of each muscle and sliced into 2 cm × 0.5 cm × 0.5 cm strips. Muscle tissue samples were stained with hematoxylin and eosin to examine the muscle fiber morphology using a biological microscope (YS100, Nikon, Tokyo, Japan) (Supplementary Fig. S1). At least 10 fibers per image were measured to determine the mean fiber diameter. The muscle fiber diameter was calculated as 2× $\sqrt{S/\pi }$, where S denotes the area of the myofiber. The muscle fiber density was counted as the total number of muscle fibers (N) in the image. The muscle fiber density was calculated as N / S_1_, where S_1_ denotes the area of each image (Chang et al., 2024). Endomysium thickness (**EMT**) was measured based on the myofiber membrane, whereas perimysium thickness (**PMT**) was measured based on the muscle bundle membrane. After correctly identifying one square millimeter field per slide, 30 measure points within the area were randomly chosen to calculate the mean EMT and PMT values, respectively (An et al., 2010; Chang et al., 2024). All measurements were calculated using ImageJ software (National Institutes of Health, Bethesda, MD, USA) (Chang et al., 2024).

### Blood collection and metabolite concentrations

Prior to slaughter at 120 d of age, 3.5 mL of blood samples were collected from the wing veins of 40 chickens (20 HRFI and 20 LRFI chickens). The serum was then transferred to 2 mL centrifuge tubes after all blood samples were centrifuged for 5 min at 4°C at 3500 rpm. Total glucose (**GLU**), creatine kinase (**CK**), creatinine (**CREA**), cholesterol (**CHOL**), triglycerides (**TG**), high-density lipoprotein cholesterol (**HDL-C**), low-density lipoprotein cholesterol (**LDL-C**), and non-esterified fatty acid (**NEFA**) were assessed via an automatic biochemical analyzer (TOSHIBA TBA-40FR, Tokyo, Japan) and commercial assay kits (Nanjing Jiancheng Bioengineering Institute, Nanjing, China).

### Statistical analysis

Statistical analysis was conducted using SPSS 27 software (IBM Corp., Armonk, NY, USA) to analyze FE traits, and RFI was calculated using the general linear regression procedure. Differences in FE traits, slaughter performance, meat quality, muscle fiber morphology, and serum biochemical indices between the HRFI and LRFI groups were compared using an independent student′s *t*-test. Spearman's coefficients between coefficients among phenotypic traits and FE were computed using Origin 2024 (OriginLab Corporation, Northampton, MA, USA). Statistical significance was set at *P* < 0.05.

## Results

### Growth performance and feed efficiency

**Fig. 1:**
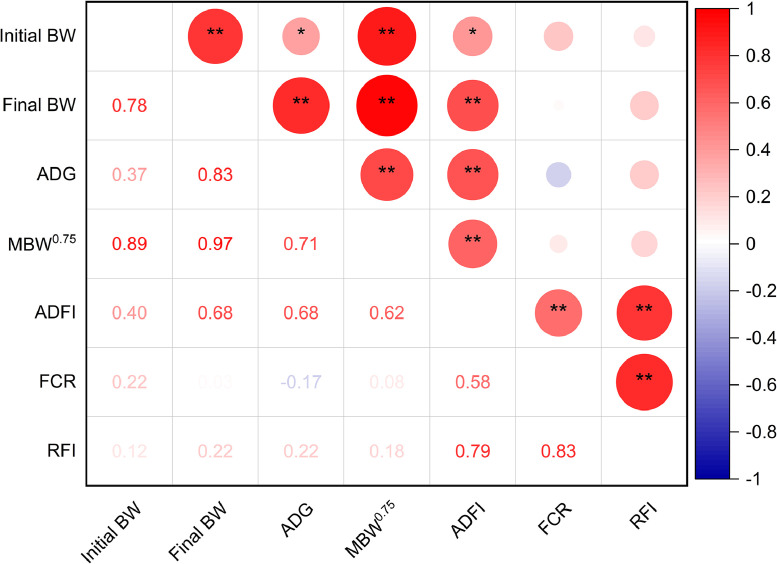
Correlation coefficients between features related to growth and FE. Red and blue gradients represent positive and negative correlation coefficients, respectively. The deeper the red or blue color, the stronger the correlation coefficient. Whiter areas indicate weaker correlations. BW, body weight; ADG, average daily gain; MBW^0.75^, metabolic body weight; ADFI, average daily feed intake; FCR, feed conversion ratio; RFI, residual feed intake. Significant differences are denoted as follows: * (*P* < 0.05), ** (*P* < 0.01).

Table 2 displays the Huaibei Partridge chickens' growth and FE data. The findings revealed that the ADFI and FCR in the HRFI group were greater than those in the LRFI group (*P* < 0.001). The LRFI and HRFI groups did not differ in their initial BW, final BW, ADG, or MBW^0.75^. RFI had a positive correlation with both ADFI (*P* < 0.001) and FCR (*P* < 0.001) based on the correlation analysis. The correlation coefficients between growth performance and FE traits are shown in Fig.1. RFI demonstrated a strong positive correlation (*P* < 0.01) with ADFI (*r* = 0.79) and FCR (*r* = 0.83), but showed no correlation with initial BW, ADG, final BW, or MBW^0.75^.

**Table 2:** Characterization of growth performance and FE between the different RFI groups of indigenous chickens.

Items^1^	HRFI^2^	LRFI	SEM^3^	*P*-value
Initial BW (g)	711.73	715.20	16.637	0.919
Final BW (g)	1428.52	1423.62	30.015	0.936
ADG (g/d)	14.34	14.17	0.410	0842
MBW^0.75^ (g/d)	186.81	186.71	2.888	0.987
ADFI (g/d)	66.04^a^	49.17^b^	1.928	<0.001
FCR (g/g)	4.63^a^	3.50^b^	0.101	<0.001
RFI (g/d)	9.24^a^	−7.29^b^	1.362	<0.001

1 BW, body weight; ADG, average daily gain; MBW^0.75^, metabolic weight; ADFI, average daily feed intake; FCR, feed conversion ratio; RFI, residual feed intake.

2 HRFI, high residual feed intake; LRFI, low residual feed intake.

3 SEM, pooled standard error of mean.

a–b Different lowercase letters on the same line indicate significant differences (*P* < 0.05).

### Carcass traits

**Fig. 2:**
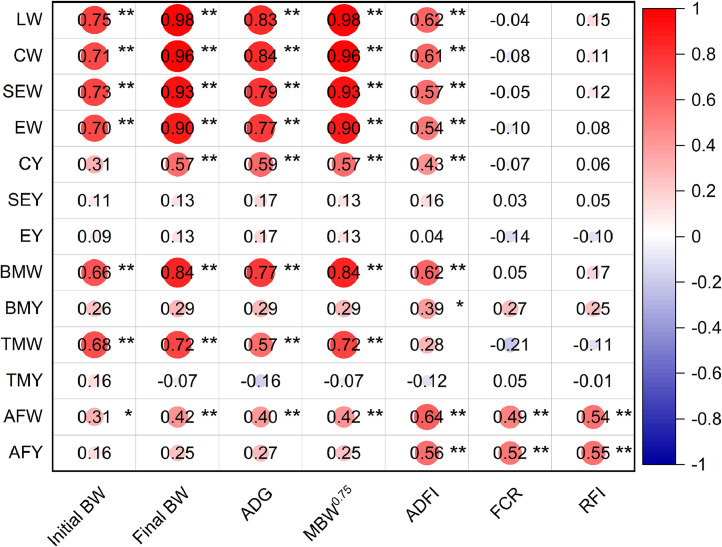
Correlation coefficients between features related to carcass traits and FE traits. Red and blue gradients represent positive and negative correlation coefficients, respectively. The deeper the red or blue color, the stronger the correlation coefficient. Whiter areas indicate weaker correlations. LW, live weight; CW, carcass weight; SEW, semi-eviscerated weight; EW, eviscerated weight; CY, carcass yield; SEY, semi-eviscerated yield; EY, eviscerated yield; BMW, breast muscle weight; BMY, breast muscle yield; TMW, thigh muscle weight; TMY, thigh muscle yield; AFW, abdominal fat weight; AFY, abdominal fat yield; BW, body weight; ADG, average daily gain; MBW^0.75^, metabolic body weight; ADFI, average daily feed intake; FCR, feed conversion ratio; RFI, residual feed intake. Significant differences are denoted as follows: * (*P* < 0.05), ** (*P* < 0.01).

Table 3 displays how RFI divergence affects carcass features. The HRFI group had a higher weight and yield of abdominal fat than the LRFI group (*P* ≤ 0.01). With the exception of AFW and AFY, the carcass traits of the HRFI and LRFI groups showed no differences. The Spearman's correlation coefficients between the carcass and FE traits are listed in Fig. 2. The findings showed a significant positive association between RFI and AFY (*P* < 0.01, *r* = 0.55) and AFW (*P* < 0.01, *r* = 0.54). FCR was positively correlated with AFY (*P* < 0.01, *r* = 0.52) and AFW (*P* < 0.01, *r* = 0.49).

**Table 3:** Effects of RFI divergence on carcass traits in indigenous chickens.

Items^1^	HRFI^2^	LRFI	SEM^3^	*P*-value
LW (g)	1386.17	1416.87	28.132	0.592
CW (g)	1239.59	1253.93	27.782	0.800
SEW (g)	1138.46	1153.65	25.475	0.770
EW (g)	1063.90	1091.12	25.170	0.595
CY (%)	89.30	88.36	0.004	0.259
SEY (%)	82.10	81.45	0.005	0.539
EY (%)	76.60	77.00	0.007	0.765
BMW (g)	59.89	56.68	1.979	0.424
BMY (%)	11.23	10.35	0.002	0.070
TMW (g)	77.18	82.76	2.119	0.191
TMY (%)	14.65	15.15	0.003	0.403
AFW (g)	10.32^a^	4.72^b^	0.883	0.001
AFY (%)	0.93^a^	0.43^b^	0.001	<0.001

1 LW, live weight; CW, carcass weight; SEW, semi-eviscerated weight; EW, eviscerated weight; CY, carcass yield; SEY, semi-eviscerated yield; EY, eviscerated yield; BMW, breast muscle weight; BMY, breast muscle yield; TMW, thigh muscle weight; TMY, thigh muscle yield; AFW, abdominal fat weight; AFY, abdominal fat yield.

2 HRFI, high residual feed intake; LRFI, low residual feed intake.

3 SEM, pooled standard error of mean.

a–b Different lowercase letters on the same line indicate significant differences (*P* < 0.05).

### Meat quality

**Fig. 3:**
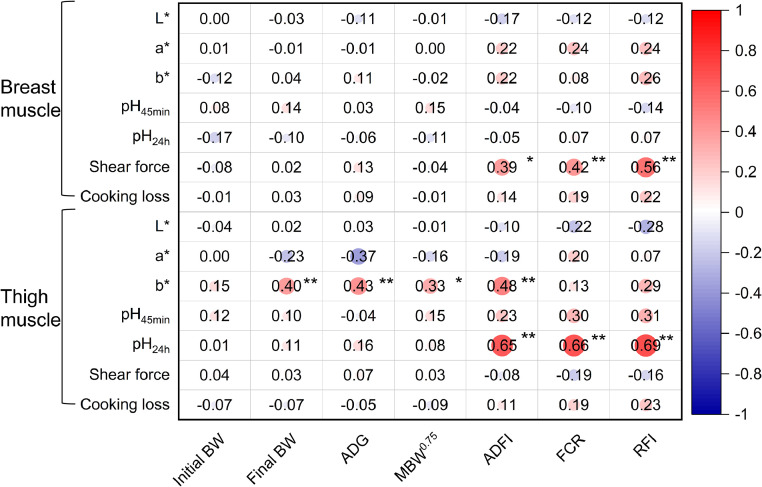
Correlation coefficients between features related to meat quality and FE traits. Red and blue gradients represent positive and negative correlation coefficients, respectively. The deeper the red or blue color, the stronger the correlation coefficient. Whiter areas indicate weaker correlations. L*, lightness; a*, redness; b*, yellowness; BW, body weight; ADG, average daily gain; MBW^0.75^, metabolic body weight; ADFI, average daily feed intake; FCR, feed conversion ratio; RFI, residual feed intake. Significant differences are denoted as follows: * (*P* < 0.05), ** (*P* < 0.01).

Table 4 shows the differences in breast and thigh meat quality traits between HRFI and LRFI chickens. Compared with the HRFI group, the LRFI group's thigh muscles exhibited lower pH_24h_ values (*P* < 0.001). In the HRFI group, the breast muscle's shear force was noticeably greater (*P* = 0.001). Additional meat quality traits also showed no discernible variations. The shear force of the breast muscle (*r* = 0.56) and the pH_24h_ values of the thigh muscle (*r* = 0.69) showed a significant positive association with RFI (*P* < 0.01; Fig. 3).

**Table 4:** Effects of RFI divergence on meat quality in indigenous chickens.

Items		HRFI^1^	LRFI	SEM^2^	*P*-value
Meat color					
Lightness (L*)	Breast muscle	51.40	53.02	0.526	0.125
	Thigh muscle	44.22	42.97	0.372	0.338
Redness (a*)	Breast muscle	5.51	5.23	0.155	0.377
	Thigh muscle	6.25	6.20	0.156	0.881
Yellowness (b*)	Breast muscle	14.50	13.38	0.378	0.139
	Thigh muscle	11.99	11.16	0.222	0.060
pH_45min_	Breast muscle	5.74	5.81	0.028	0.251
	Thigh muscle	6.16	6.07	0.032	0.174
pH_24h_	Breast muscle	5.61	5.59	0.019	0.601
	Thigh muscle	6.03^a^	5.82^b^	0.023	<0.001
Shear force (N)	Breast muscle	29.99^a^	24.19^b^	0.929	0.001
	Thigh muscle	30.12	34.55	1.609	0.171
Cooking loss (%)	Breast muscle	21.94	19.84	0.007	0.112
	Thigh muscle	24.46	22.30	0.009	0.216

1 HRFI, high residual feed intake; LRFI, low residual feed intake.

2 SEM, pooled standard error of mean.

a–b Different lowercase letters on the same line indicate significant differences (*P* < 0.05).

### Muscle fiber morphology

**Fig. 4:**
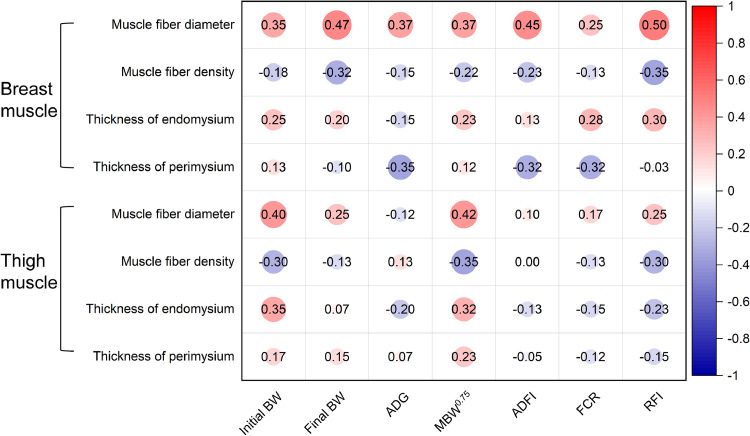
Correlation coefficients between features related to muscle fiber morphology traits and FE traits. Red and blue gradients represent positive and negative correlation coefficients respectively. The deeper the red or blue color, the stronger the correlation coefficient. Whiter areas indicate weaker correlations. BW, body weight; ADG, average daily gain; MBW^0.75^, metabolic body weight; ADFI, average daily feed intake; FCR, feed conversion ratio; RFI, residual feed intake. Significant differences are denoted as follows: * (*P* < 0.05), ** (*P* < 0.01).

RFI and muscle fiber morphological traits did not significantly differ (Table 5). The correlation coefficients between FE and muscle fiber morphology traits are shown in Fig. 4. The findings indicated that there was no correlation between the RFI and characteristics of muscle fiber morphology.

**Table 5:** Effects of RFI divergence on muscle fibers in indigenous chickens.

Items	Sample	HRFI^1^	LRFI	SEM^2^	*P*-value
Muscle fiber diameter (μm)	Breast muscle	37.38	34.11	1.576	0.324
	Thigh muscle	39.27	37.87	1.031	0.528
Muscle fiber density (number/mm^2^)	Breast muscle	717.83	758.95	37.617	0.614
	Thigh muscle	697.23	722.58	35.868	0.746
Thickness of endomysium (μm)	Breast muscle	4.91	5.43	0.268	0.357
	Thigh muscle	6.36	5.71	0.415	0.466
Thickness of perimysium (μm)	Breast muscle	21.9	24.39	1.177	0.318
	Thigh muscle	25.56	29.63	1.216	0.094

1 HRFI, high residual feed intake; LRFI, low residual feed intake.

2 SEM, pooled standard error of mean.

### Blood biochemical parameters

**Fig. 5:**
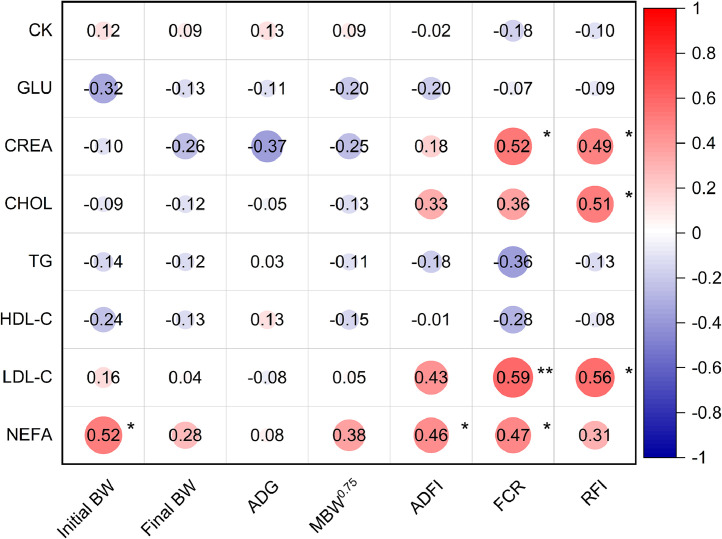
Correlation coefficients between features related to blood biochemical parameters and FE traits. Red and blue gradients represent positive and negative correlation coefficients, respectively. The deeper the red or blue color, the stronger the correlation coefficient. Whiter areas indicate weaker correlations. Glu, glucose; CK, creatine kinase; CREA, creatinine; CHOL, cholesterol; TG, triglyceride; HDL-C, high density lipoprotein cholesterol; LDL-C, low density lipoprotein cholesterol; NEFA, non-esterified fatty acids; BW, body weight; ADG, average daily gain; MBW^0.75^, metabolic body weight; ADFI, average daily feed intake; FCR, feed conversion ratio; RFI, residual feed intake. Significant differences are denoted as follows: * (*P* < 0.05), ** (*P* < 0.01).

Table 6 presents the effects of RFI divergence on blood biochemical markers. The findings indicate that the HRFI group had greater levels of CREA, CHOL, LDL-C, and NEFA (*P* < 0.05). Additionally, no significant differences were observed between RFI and other blood biochemical markers. Serum levels of CREA (*r* = 0.49), CHOL (*r* = 0.51), and LDL-C (*r* = 0.56) illustrated a moderately positive correlation with the RFI (*P* < 0.05; Fig. 5).

**Table 6:** Effects of RFI divergence on blood biochemical parameters in indigenous chickens.

Items^1^	HRFI^2^	LRFI	SEM^3^	*P*-value
GLU (mmol/L)	13.95	14.51	0.651	0.678
CK (U/L)	2897.10	2951.50	71.844	0.716
CREA (μmol/L)	19.20^a^	13.40^b^	1.350	0.033
CHOL (mmol/L)	5.05^a^	4.41^b^	0.140	0.017
TG (mmol/L)	0.43	0.49	0.026	0.267
HDL-C (mmol/L)	2.44	2.51	0.053	0.581
LDL-C (mmol/L)	1.83^a^	1.29^b^	0.112	0.011
NEFA (mmol/L)	0.94^a^	0.60^b^	0.079	0.025

1 Glu: glucose; CK: creatine kinase; CREA: creatinine; CHOL: cholesterol; TG: triglyceride; HDL-C: high density lipoprotein cholesterol; LDL-C: low density lipoprotein cholesterol; NEFA: non-esterified fatty acids.

2 HRFI, high residual feed intake; LRFI, low residual feed intake.

3 SEM, pooled standard error of mean.

a-b Different lowercase letters on the same line indicate significant differences (*P* < 0.05).

## Discussion

This study investigated the influence of divergence in RFI on growth performance, carcass traits, meat quality, muscle fiber morphology, and blood chemistry in indigenous chickens. Our results verified that RFI was a substantial correlation between ADFI and FCR. However, RFI did not correlate with either initial BW, final BW, MBW^0.75^, or ADG. Similar findings have been reported in small-sized meat ducks (Li et al., 2023b), fast-growing meat ducks (Liu et al., 2024), and other indigenous chicken breeds (Yang et al., 2020; Yi et al., 2018). These findings suggest that selecting for lower RFI can maintain BW, improving FE in indigenous chicken breeds.

Greater FE is shown by LRFI, which enables animals to grow at comparable rates while using less feed (Yi et al., 2018). Consistent with this, our data showed that LRFI birds consumed less feed without compromising BW. Furthermore, the HRFI and LRFI chickens in this investigation did not differ significantly in terms of any other carcass traits. However, AFW and AFY were much lower in the LRFI group, showing a substantial positive correlation with RFI. These findings are supported by earlier research on local chicken breeds (Yang et al., 2020) and broilers (Zerehdaran et al., 2004). Research has shown that abdominal fat in poultry primarily accumulates around the cloaca and gizzard (Claire D'Andre et al., 2013; Nematbakhsh et al., 2021), and excessive adiposity not only reduces FE but also conflicts with consumer preferences for leaner meat (Claire D'Andre et al., 2013). Therefore, selecting chickens with LRFI values could enhance FE while reducing fat deposition.

As economic development accelerates and living standards rise, consumers demand for higher-quality meat continues to grow. Flavor and nutritional value play a crucial role in meat selection. Since meat quality is assessed using multiple indices, we selected meat color, pH, shear force, and cooking loss as key evaluation criteria. Meat pH, an important indicator of freshness and edibility, reflects post-slaughter biochemical changes. Consistent with other research, our findings demonstrated that the LRFI group's thigh muscle pH_24h_ value was lower than that of the HRFI group (Yang et al., 2020). Additionally, a study on how RFI affects muscular energy metabolism and meat quality in pigs revealed that choosing low RFI reduces the pH in lean meat (Faure et al., 2013). However, other studies have shown that RFI does not affect pH (Bai et al., 2022; Li et al., 2023b). The aforementioned studies demonstrate that the correlations between RFI and pH were inconsistent, which were mainly due to different breeds or experimental conditions.

Meat tenderness is a key factor influencing consumer preference. In this study, shear force analysis revealed that the breast muscle in LRFI chickens was softer than that in HRFI chickens, consistent with previous findings in small-sized ducks (Bai et al., 2022). However, a study in sheep indicated a negative correlation between shear force and RFI (Gurgeira et al., 2022). Another study on indigenous chickens showed no statistically significant differences in shear force associated with RFI (Yang et al., 2020). Hence, the correlations between RFI and shear force are affected by breed variations or experimental conditions. Moreover, regarding other meat quality traits, no significant correlation with RFI was found, consistent with prior studies on chickens (Wen et al., 2018; Yang et al., 2020) and ducks (Li et al., 2023b; Liu et al., 2024).

Although RFI is a key measure of FE, selecting chickens with high FE in commercial production is both time-consuming and labor-intensive. To address this, researchers have explored blood metabolites linked to RFI, reducing reliance on costly labor, specialized equipment, and lengthy feed trials. Notably, several researchers have studied the relationship between FE and plasma metabolites (Bai et al., 2022; Clemmons et al., 2023; Yang et al., 2020). This study investigated the association between RFI and blood biochemical indicators to facilitate the selection of high feed efficiency chickens.

CREA level is a marker of renal function and is influenced by skeletal muscle mass (Kashani et al., 2018; Lin et al., 2021; Nishiki et al., 2021; Osaka et al., 2018; Romeo et al., 2021). A reduction in muscle mass typically leads to reduced serum creatinine levels (Viollet et al., 2009). Our results indicated a positive correlation between plasma CREA concentration and RFI, consistent with a previous study reporting elevated CREA levels in HRFI male Sahiwal calves (Baban et al., 2021). This suggests that HRFI chickens may produce more CREA owing to surplus nutrition and distinct metabolic processes compared with LRFI chickens.

CHOL is considered a biomarker of metabolic disorders and a precursor of steroid hormones, bile acids, and vitamin D (Hajjar et al., 2019). Furthermore, cholesterol level is a potential predictor of RFI (Metzler-Zebeli et al., 2017). Aligning with earlier studies on indigenous chickens that found a moderate link between RFI and cholesterol, the results of this investigation showed that the concentrations of cholesterol in HRFI chickens were higher than those in LRFI chickens (Yang et al., 2020). Therefore, cholesterol can be used as an indicator of FE in broilers. In poultry, elevated LDL-C levels indicate lipid metabolism disorders and may contribute to metabolic diseases (Li et al., 2023a). In contrast, HDL-C helps clear LDL-C by transporting it to the liver for metabolism (Zhang et al., 2023). In this study, LDL-C concentrations were higher in the HRFI group, and RFI moderately correlated with LDL-C levels. This is consistent with prior studies on indigenous chickens (Yang et al., 2020) and supports the hypothesis that HRFI chickens consume more energy, which is subsequently stored as abdominal fat. The higher abdominal fat weight and yield observed in HRFI chickens in this study support this conclusion, indicating that LDL-C content can be utilized as a predictor of broiler FE.

NEFAs serve as a primary lipid source for various tissues and can be broken down into free fatty acids, which contribute to gluconeogenesis. This result is consistent with other studies on growing beef heifers, which found HRFI animals had higher NEFA concentrations (Kelly et al., 2010). This suggests LRFI chickens may utilize NEFAs more efficiently for energy production, thereby reducing fat deposition. Overall, these results suggest serum metabolites can serve as cost-effective predictors of RFI.

## Conclusion

This study examined the influence of RFI divergence on growth performance, carcass traits, meat quality, muscle fiber morphology, and blood chemistry in indigenous chickens. Our findings indicate that targeting LRFI enhances feed efficiency in indigenous chickens without affecting their BW and muscle fiber morphology. Furthermore, selecting an appropriate LRFI value for chickens is helpful in reducing fat deposition. Moreover, the levels of CREA, CHOL, and LDL-C in indigenous chicken breeds can function as indirect selection indicators of FE. Further studies are necessary to investigate and validate the effects of CREA, CHOL, and LDL-C on FE in future breeding programs.

## Availability of data and materials

None of the data were deposited in an official repository. Data can be obtained from the authors upon reasonable request.

## Supplementary information

Supplementary Fig. S1. The myofiber characteristics of indigenous chickens between the HRFI and LRFI groups were examined using hematoxylin and eosin staining.

## CRediT authorship contribution statement

**Wenjing Chen:** Writing – original draft, Visualization, Validation, Methodology, Formal analysis, Data curation. **Weiqi Wang:** Writing – original draft, Methodology, Data curation. **Xin Wang:** Visualization, Validation, Resources, Formal analysis, Data curation. **Xuling Liu:** Validation, Methodology, Formal analysis, Data curation. **Liang Chang:** Validation, Methodology, Formal analysis, Data curation. **Haoming Chang:** Validation, Formal analysis, Data curation. **Yunxia He:** Visualization, Formal analysis. **Zhaoyu Geng:** Writing – original draft, Methodology, Funding acquisition, Conceptualization. **Sihua Jin:** Writing – review & editing, Writing – original draft, Visualization, Methodology, Funding acquisition, Conceptualization.

## Disclosures

The authors declare that they have no competing interests.

## Appendix Supplementary materials

Image, application 1

Image, application 2
