# Supplementary material for: Influence of divergence in residual feed intake on growth performance, carcass traits, meat quality, muscle fiber morphology, and blood chemistry in indigenous chickens
Source: Poult Sci. 2025 Aug 20;104(11):105712. doi: 10.1016/j.psj.2025.105712 (PMC12683118; doi:10.1016/j.psj.2025.105712)
Supplement: Supplementary file 1 [file mmc1.docx]

**Influence of divergence in residual feed intake on growth performance, carcass traits, meat quality, muscle fiber morphology, and blood chemistry in indigenous chickens**

Wenjing Chen^1,2^, Weiqi Wang^1,2^, Xin Wang^1,2^, Xuling Liu^1,2^, Liang Chang^1,2^, Haoming Chang^1,2^, Yunxia He^1^, Zhaoyu Geng^1,2^, Sihua Jin^1,2,*^

^1^College of Animal Science and Technology, Anhui Agricultural University, Hefei 230036, China

^2^Anhui Provincial Key Laboratory of Local Animal Genetic Resources Conservation and Bio-breeding, Hefei 230036, China

* Correspondence: Dr. and associate Prof. Sihua Jin, College of Animal Science and Technology, Anhui Agricultural University, Hefei 230036, China.

Tel:+86-551-6578 6357

Fax:+86-551-6578 6328

E-mail: [jsh3235@126.com](mailto:jsh3235@126.com)

Section: Management and Production


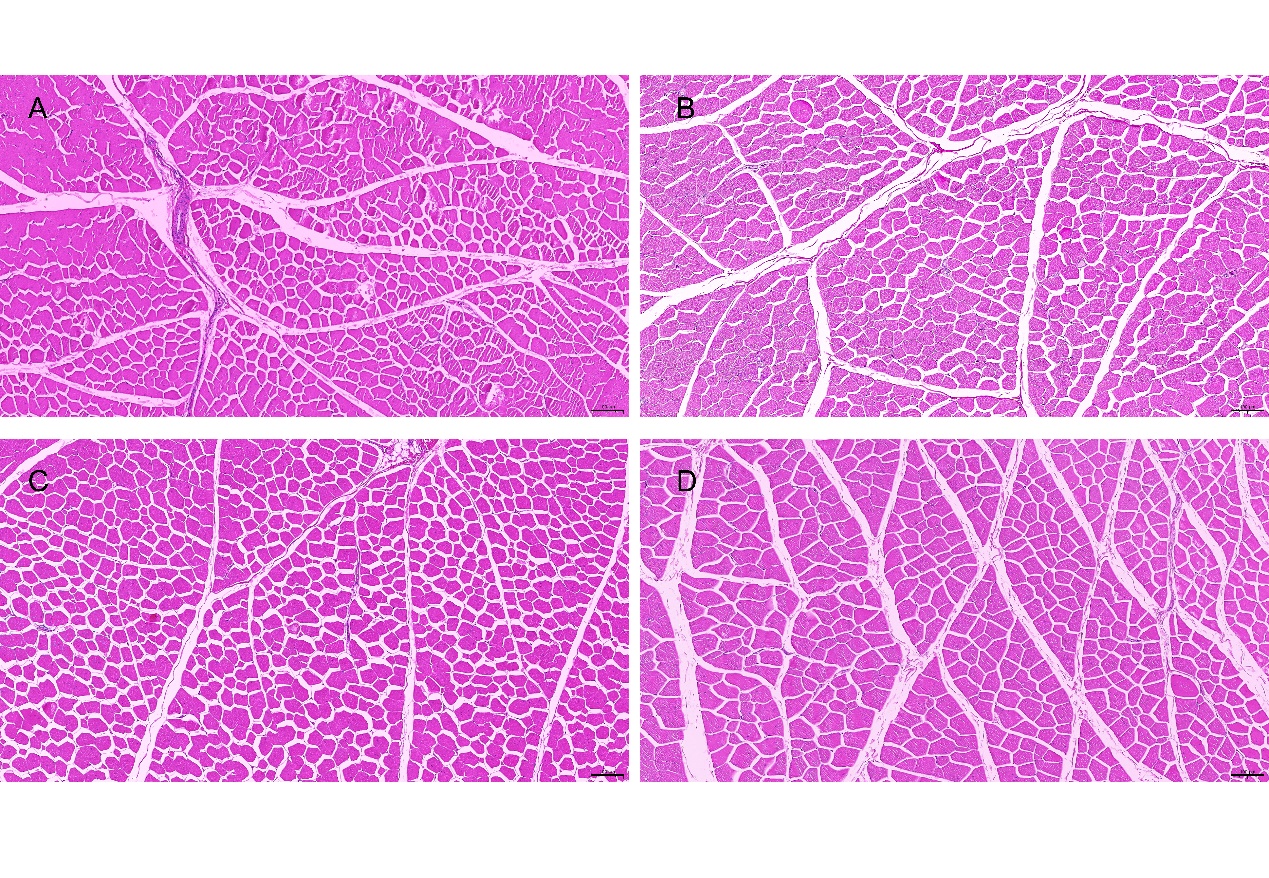


**Supplementary Fig. S1**. The myofiber characteristics of indigenous chickens between the HRFI and LRFI groups were examined using hematoxylin and eosin staining. A. Myofiber characteristics of the breast muscle in the LRFI group. B. Myofiber characteristics of the thigh muscle in the LRFI group. C. Myofiber characteristics of the breast muscle in the HRFI group. D. Myofiber characteristics of the thigh muscle in the HRFI group. Magnification of 100× was used.
