# Supplementary material for: Influence of divergence in residual feed intake on growth performance, carcass traits, meat quality, muscle fiber morphology, and blood chemistry in indigenous chickens
Source: Poult Sci. 2025 Aug 20;104(11):105712. doi: 10.1016/j.psj.2025.105712 (PMC12683118; doi:10.1016/j.psj.2025.105712)
Supplement: Supplementary file 2 [file mmc2.pdf]

editage

# Editing Certificate

This document certifies that the manuscript listed below has been edited to ensure language and grammar accuracy and is error free in these aspects. The logical presentation of ideas and the structure of the paper were also checked during the editing process. The edit was performed by professional editors at Editage, a brand of Cactus Communications. The author's core research ideas were not altered in any way during the editing process. The quality of the edit has been guaranteed, with the assumption that our suggested changes have been accepted and the text has not been further altered without the knowledge of our editors.

MANUSCRIPT TITLE

**Influence of divergence in residual feed intake on growth performance, carcass traits, meat quality, muscle fiber morphology, and blood chemistry in indigenous chickens**

AUTHORS

**Wenjing Chen, Weiqi Wang, Xin Wang, Xuling Liu, Liang Chang, Haoming Chang, Yunxia He, Zhaoyu Geng, Sihua Jin***

ISSUED ON

**July 21, 2025**

JOB CODE

**GAOJI_33_3**

THE EDITAGE Promise QUALITY GUARANTEE

Prabh Grewal
Senior Vice President - Editage

editage | helping you get published

Since 2002, Editage has helped over 430,000 authors publish around 1.2 million research papers in scholarly journals across over 1000 disciplines through editorial, translation, transcription, and publication support services. Editage is a brand of Cactus Communications (cactusglobal.com), a science communication and technology company.

CACTUS.

GLOBAL :
+1(669) 272-1214 | request@editage.com

CHINA :
400-001-8237; 021-60209400 | fabiao@editage.cn
